# Dynamics of affect modulation in neurodevelopmental disorders (DynAMoND) – study design of a prospective cohort study

**DOI:** 10.1186/s40345-024-00367-2

**Published:** 2024-12-28

**Authors:** Maximilian Bayas, Tobias D. Kockler, Josep Antoni Ramos-Quiroga, Silvia Muñoz Caller, Christian Fadeuilhe, Giovanni de Girolamo, Laura Iozzino, Miriam D’Addazio, Jan Haavik, Anne Halmøy, Karin Schiøler Hellum, Joakim Njaastad Kolle, Berge Osnes, Astri J. Lundervold, Nader Perroud, Roland Hasler, Mélanie Teixeira De Almeida, Ulrich W. Ebner-Priemer, Sharmili Edwin Thanarajah, Carmen Schiweck, Silke Matura, Jonathan Repple, Andreas Reif, Mareike Aichholzer

**Affiliations:** 1 Department for Psychiatry, Psychosomatic Medicine and Psychotherapy, University Hospital Frankfurt-Goethe University, Frankfurt am Main, Germany; 2 Mental mHealth Lab, Karlsruhe Institute of Technology, Karlsruhe, Germany; 3 Department for Psychiatry, Hospital Universitari Vall d’Hebron, Barcelona, Catalonia Spain; 4 Group of Psychiatry, Mental Health and Addictions, Vall d’Hebron Research Institute (VHIR), Barcelona, Catalonia Spain; 5 Biomedical Network Research Centre on Mental Health (CIBERSAM), Barcelona, Catalonia Spain; 6 Epidemiological and Evaluation Psychiatry Unit, IRCCS Istituto Centro San Giovanni di Dio Fatebenefratelli, Brescia, Italy; 7 K.G. Jebsen Centre for Neuropsychiatric Disorders, Department of Biomedicine, University of Bergen, Bergen, Norway; 8 Division of Psychiatry, Haukeland University Hospital, Bergen, Norway; 9 Department of Clinical Psychology, University of Bergen, Bergen, Norway; 10 Department of Biological and Medical Psychology, University of Bergen, Bergen, Norway; 11 Department of Clinical Medicine, University of Bergen, Bergen, Norway; 12 Department of Psychiatry, Faculty of Medicine, University of Geneva, Geneva, Switzerland; 13 TRE Unit, Division of Psychiatric Specialties, Department of Psychiatry, University Hospital of Geneva, Geneva, Switzerland; 14 Institute for Translational Psychiatry, Epidemiological and Evaluation Psychiatry, University of Münster, Münster, Germany

**Keywords:** Attention-deficit/hyperactivity disorder, Borderline personality disorder, Bipolar disorder, Ambulatory assessment, Digital phenotyping, Affective state, Emotion dysregulation

## Abstract

**Background:**

Attention-deficit/hyperactivity disorder (ADHD) is a common neuro-developmental disorder that often persists into adulthood. Moreover, it is frequently accompanied by bipolar disorder (BD) as well as borderline personality disorder (BPD). It is unclear whether these disorders share underlying pathomechanisms, given that all three are characterized by alterations in affective states, either long or short-term. BD is characterized by infrequent but intense mood shifts, while ADHD and BPD involve more dynamic emotional fluctuations. It is yet to be determined whether these disorders represent distinct phenomena or different points on a spectrum of affective dysregulation.

**Methods:**

This study seeks to distinguish the emotional dysregulation of BPD, ADHD, and BD by using digital phenotyping, a measurement burst electronic-diary method with different sampling rates, and accelerometry to measure participants’ activity. Our study will include 480 participants aged 14 to 50 (120 each from BPD, ADHD, BD, and healthy control groups) from five European sites. Participants’ smartphones will provide continuous data on their digital phenotypes, i.e., by indicators of physical activity and communication, for one year, along with daily evening ratings of mood and sleep. Moreover, five intensive measurement periods of five days each, called measurement bursts, will occur throughout the year, with electronic diaries asking participants to report on mood, self-esteem, impulsivity, life events, social interactions, and dysfunctional behaviors ten times a day. Moreover, participants will wear activity sensors during the five measurement bursts. Statistical analysis aims to identify whether affective dysregulation aspects share or differ across disorders. Specifically, data analysis aims to investigate the differences in parameters of affect fluctuation such as attractor strength and variability between disorders and to test the association of genetic risk factors for psychiatric disorders and resilience factors with critical parameters of affect modulation.

**Discussion:**

The results of this study offer the potential to link patients’ external exposures with their affective state, reduce misdiagnosis, and determine the best timing for therapeutic interventions. Potential limitations of the study include insufficient recruitment of patients and drop-outs due to various protocol violations.

**Trial registration:**

Study code: DRKS00028917, registered 27.07.2022, https://drks.de/search/de/trial/DRKS00028917.

## Background

Neuropsychiatric disorders, such as bipolar disorder (BD), borderline personality disorder (BPD), and attention-deficit/hyperactivity disorder (ADHD), are prevalent conditions that typically begin in childhood or adolescence and persist into adulthood. All three disorders have a developmental origin, with genetic research suggesting that ADHD and BD are lying on a neurodevelopmental continuum [1]. According to the diagnostic criteria [2], the core symptoms of ADHD manifest before age 12, whereas BPD and BD often emerge during adolescence or early adulthood. BD is defined by the first full mood episode, which typically occurs in early adulthood, but it may have developmental precursors in childhood [3]. In the early stages of BD, non-episodic symptoms like mood irregularities, anxiety, and sleep disturbances may be present, often termed “bipolar prodrome” [4, 5], making it difficult to differentiate it from ADHD or emerging BPD. All three disorders involve varying degrees of affect fluctuations that can be chronic and severe.

ADHD is the most common and earliest neurodevelopmental disorder, affecting 5–7% of children [6, 7]. In adulthood, 15% with a childhood ADHD diagnosis still meet the complete diagnostic criteria for ADHD, while approximately 60% continue to experience debilitating symptoms in adulthood [6–8]. Adults with ADHD are at significant risk for negative outcomes, such as accidents [9], suicide [10], and overall mortality [6], thus indicating a possible negative health trajectory [11, 12]. As ADHD symptoms evolve, with a decrease in hyperactivity and an increase in affective fluctuations [13, 14], there is a potential overlap with BD, a disorder which is characterized by fluctuating moods [15]. Recent research supports this hypothesis and suggests that approximately 8% of adults with ADHD also have BD, while 17% of adults with BD also have ADHD, resulting in a prevalence rate of 0.4% for the comorbid condition [16]. Family-based studies have also provided evidence of a joint genetic contribution between ADHD and BD, with a cross-disorder GWAS meta-analysis further supporting this hypothesis [17]. However, misdiagnosis and mistreatment can occur due to the similarity of symptoms between BD and ADHD, particularly in individuals with rapid cycling courses, mixed episodes, and in those with bipolar-II disorder, which is diagnosed by at least one episode of major depression and at least one hypomanic episode, and which is commonly characterized by impulsivity [2].

Numerous studies have shown that there is a significant comorbidity between ADHD and BPD [18–21]. Approximately 25% of adults with ADHD meet the diagnostic criteria for BPD, and at least 14% of individuals who later received a diagnosis of BPD had childhood ADHD [18]. Additionally, retrospective studies suggest that 60% of patients with BPD have had symptoms indicative of childhood ADHD [19]. ADHD is therefore considered a strong risk factor for the development of BPD [20], as it may increase the odds of exposure to stressful life events during childhood, which is the most robust risk factor for BPD [21]. In addition to environmental factors, ADHD and BPD share genetic vulnerability, demonstrated in both twin studies and GWAS studies [22]. GWAS studies also present evidence of genetic overlap between BD and BPD [23] and meta-analyses argue for mutual comorbidity of around 20% [24]. It should be noted that the symptoms overlap significantly across all three disorders, commonly leading to misdiagnosis. Depression is another common factor in all the studied disorders: Bipolar disorder requires a depressive episode for diagnosis [2], ADHD [25] and BD [26] patients also have high depression rates. This overlap too often complicates the diagnostic process.

ADHD, BPD, as well as BD, are characterized by difficulties in affect regulation starting early in development. BD is characterized by the episodic occurrence of mania or depression, long-lasting mood episodes with little within variation, separated by variably long phases of euthymia with mood episodes that can be indistinguishable from those experienced by HCs [2]. On the other hand, in ADHD and BPD, patients have difficulties in regulating their emotions [27]. This results in short-term affect fluctuations in patients with ADHD and BPD, often triggered by external stimuli, with high frequency and relatively low amplitude, that differ from a balanced mood. This does not necessarily mean that they have to meet the criteria for an affective episode according to ICD-10 or DSM-5. However, this seemingly clear distinction is blurred by the fact that both types of affective symptoms, such as on the one hand mood episodes meeting ICD-10 or DSM-5 criteria (depression or mania in BD and comorbid depressive episodes in ADHD and BPD patients) and on the other hand affective fluctuations with high frequency and low amplitude, can be found within one person. Furthermore, empirical data that distinguishes between these phenomena is lacking.

**Fig. 1:**
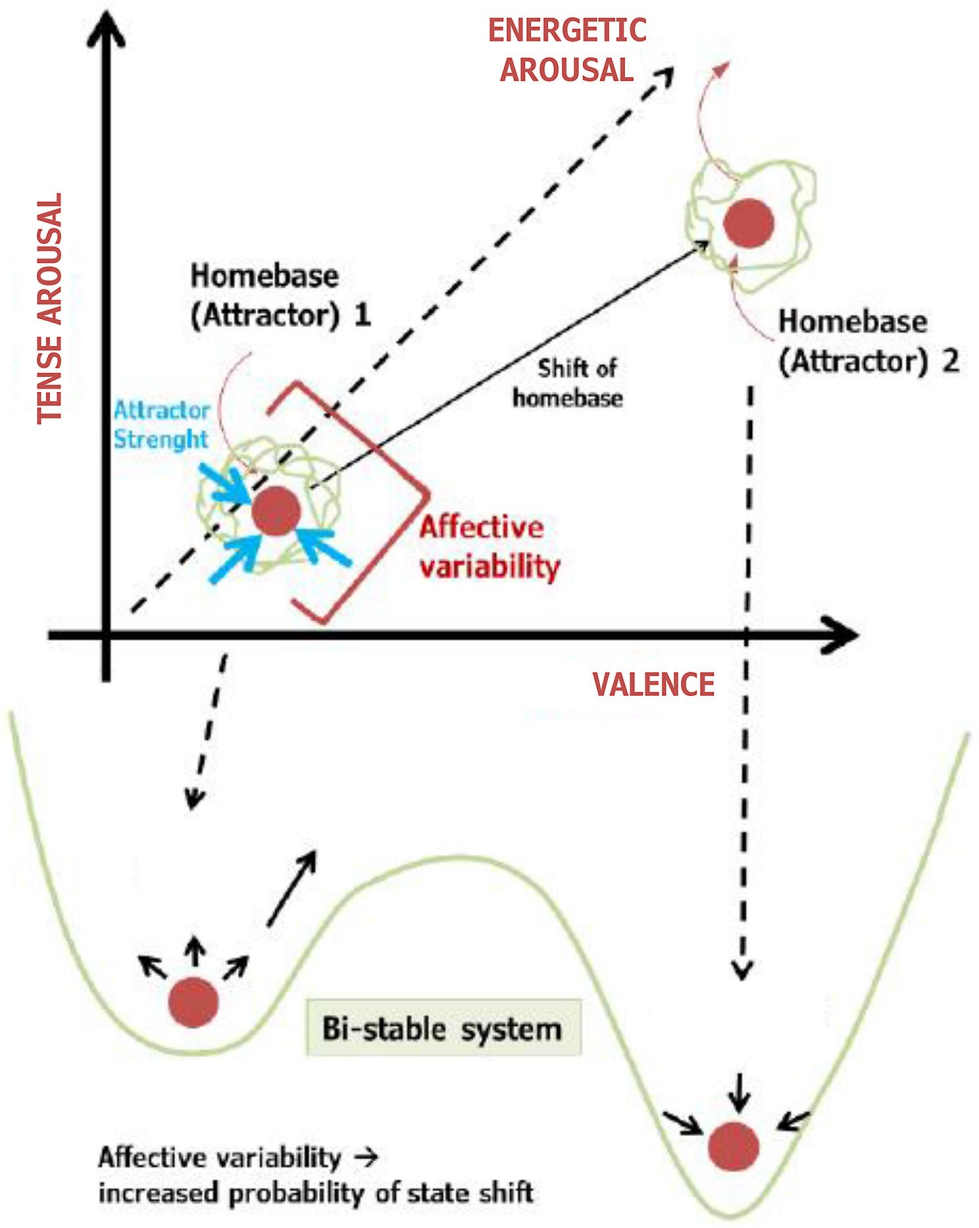
Sketch of the modified DynAffect Model (modified from [30]) using the example of a phase transition from a depressive to a manic episode, which is characterized by a shift in the affective homebase (= attractor). Both the original homebase and the shifted homebase (= mood episode) can be interpreted as two separate states in a bi-stable system exhibiting a double-well potential, i.e., the transition between the two states requires increased variability to enable the shift to the other stable state. In major mood episodes (especially in BD), variability is lower and attractor strength is higher so that the transition probability to the “normal” homebase is low. The red dots represent homebases, that can also include episodes such as mania or depression. The green lines around them resemble the current affective fluctuations, i.e. a more temporary construct.

Dynamic systems theory, originally a mathematical concept, has been applied in psychology to model the intricate dynamics of behavioral and cognitive processes [28]. It introduces the notions of attractors, stable states towards which systems tend to return, and transients, temporary states experienced before reaching an attractor [28, 29]. Building upon dynamic systems theory, the DynAffect Model [30] offers insight into individual differences in the temporal patterns and trajectories of affect dynamics, furthering our understanding of affect regulation across different psychological disorders. The DynAffect Model postulates three key dynamical properties: homebase, affective variability, and attractor strength. The homebase represents the attractor of the dynamic system and can be characterized by the predominance of positive or negative moods. Affective variability refers to the amount of reactivity to internal and external events, while attractor strength reflects how rigidly affective variability is regulated back to the attractor (Fig. 1). The DynAffect Model was originally developed in basic affective science, concerning non-clinical populations, where variability is seen as a positive feature reflecting a broad affective spectrum, but the impact of excessive variability is not well-known. In conditions such as BPD and ADHD, such excessive variability is the dominant phenomenon, often labelled as “affective dysregulation”.

Applied to psychopathology, we refer to these two stable wells, for example, depressive episode or remitted state, as homebases. The momentary affective state of a patient oscillates around these homebases and is regulated back to them after variable changes. Extending the DynAffect Model, we propose that patients with affective disorders (including ADHD and BPD patients with depressive episodes) are characterized by multiple homebases. During major mood episodes, such as depression or mania, patients move to another homebase and return to its original position upon remission. This suggests that affective dynamics might operate as a bistable system, with depression and remission resembling double-well potentials with reduced variability during depression. This concept, inspired by principles from dynamic systems theory, illustrates energy landscapes with a barrier separating two stable states. In psychopathology, these stable states, such as depressive episodes or a longer lasting affective state that does not fulfil the criteria of an affective (manic/depressive) episode, are referred to as homebases. In simple terms, an affective homebase can be seen as a stable state representing an individual’s average emotional baseline, around which different affective fluctuations occur. Excessive variability increases the likelihood of transitioning between these homebases, or attractors, in such a bistable system.

There are several clinical assumptions or speculations about altered affect dynamics in different disorders. For instance, it is suggested that the ability to control and regulate affective variability and attractor strength is primarily disturbed in ADHD and BPD. Additionally, in BD, the transition between mood states may occur due to bottom-up processes, regardless of variability and strength. However, the precise neurobiological mechanisms underlying these dynamics remain unclear. To test these hypotheses, a valid and unbiased measure of affect fluctuations over time and considerations of external stressors are needed.

To study affective dynamics in patient populations, Ambulatory Assessment (AA) is the method of choice as it enables the real-time collection of within-person data in the real world using electronic diaries [31]. It is, moreover, now increasingly complemented by continuous and more objective mobile sensing data, enabling digital phenotyping [32]. To date, prior AA studies successfully applied the DynAffect Model in patients with BPD [33, 34] or investigated specific features of affect dynamics, such as instability of affect and self-esteem (for example [35–37]), or inertia [38] across several clinical groups. Furthermore, recent studies used other statistically advanced approaches to Model the affect dynamics in patients with major depression [39, 40] or empirically analyzed phase transitions using early warning signs in affective disorders [41, 42]. However, studies that (a) include ADHD or BD samples, (b) capture longer assessment periods, (c) combine multimethod data sources, and (d) include digital phenotyping data, are lacking.

## Design

### Objectives

Our main objectives are (a) to characterize all components of the modified DynAffect Model, namely home-bases, variability, and attractor strength (shared and specific) in ADHD, BD, and BPD, (b) to investigate the influence of stressor exposure (both macro- and micro-stressors, measured with high granularity) and sleep therein, and (c) to examine the contribution of polygenic risk factors for depression, ADHD, BD, BPD, neuroticism, and resilience therein. To carry out these objectives, we will carefully characterize patients clinically, and densely measure affect fluctuations using AA and established tools.

### Hypotheses

1. Patients with BPD and ADHD show increased variability and lower attractor strength (i.e., a slower return to baseline) in valence, tense arousal, and energetic arousal compared to patients with BD.
2. In depressive (or manic) episodes, patients transition between their multiple affective homebases in all three disorders; i.e., during a depressive episode, patients predominantly reside in a homebase characterized by lower valence, higher tense arousal, and lower energetic arousal compared to a remitted (non-depressive, non-manic) episode. This refers to patients with bipolar disorder, as well as those with ADHD and those with BPD who experience depressive episodes. Conversely, during a manic episode, patients predominantly occupy a homebase marked by higher valence, higher tense arousal, and higher energetic arousal compared to a remitted (non-depressive, non-manic) episode.
3. Environmental factors such as stress or sleep have a larger effect on triggering affective fluctuations in patients with ADHD and those with BPD compared to patients with BD and HC.
4. Polygenic risk scores (PRS) for depression, mania, and BD are associated with an increased likelihood for a shift of the affective homebase, while PRS for BPD, ADHD, and neuroticism are associated with increased affective variability and decreased attractor strength, while the opposite is the case for Resilience PRS.

Sex and menstrual cycle will be accounted for as a covariate (moderating factor) in all analyses.

## Methods

This study protocol was prepared according to the SPIRIT statement guideline to ensure a high quality of describing the study [43]. The study protocol, patient/healthy participant information, consent for minors and adults were approved by the Ethics Committee of the coordinating center University Hospital Frankfurt, Germany (reference: 2022 − 645) on June 29th, 2022, and all the participating centers. The most recent version of the protocol (Version 5) was approved on February 1st, 2024. Written informed consent of the participants’ legal caretakers (for minors) and written informed consent of the participants themselves are required for participation in the study.

### Participating sites

DynAMoND is an international, Europe-wide prospective cohort study to investigate shared and unique aspects of affect fluctuation across the disorders ADHD, BD, and BPD as well as healthy volunteers. Participants will be recruited at the IRCCS Istituto Centro San Giovanni di Dio Fatebenefratelli (FBF), Unit of Epidemiological and Evaluation Psychiatry, Brescia (Italy), Vall d’Hebron Research Institute (VHIR), Group of Psychiatry, Addiction, and Mental Health, Barcelona (Spain), University Hospitals of Geneva (UNIGE-HUG), TRE Unit, Service of Psychiatric Specialties, Department of Psychiatry (Switzerland), University of Bergen and Division of Psychiatry (UiB), Haukeland University Hospital (Norway), and Department of Psychiatry, Psychosomatic Medicine and Psychotherapy, Goethe University Frankfurt (Germany) (GUF). By including multiple sites, we ensure the recruitment of a sufficient number of participants. It also offers the opportunity to prove the validity of the studied models across countries.

### Study subjects

To characterize affective variability and to investigate whether it differs between ADHD, BD, and BPD, we will study four groups: (1) adolescent/adult ADHD patients, (2) early course BD patients, (3) young patients suffering from BPD and (4) matched HC, 120 subjects of each group (Table 1).

**Table 1:** Numbers of patients to be recruited by each study site:

Disorder	GUF	VHIR	UiB	FBF	UNIGE-HUG	Sum
ADHD	24	24	24	24	24	120
BD	24	24	24	24	24	120
BPD	24	24	24	24	24	120
HC	24	24	24	24	24	120

### Inclusion criteria

All participants will undergo the Mini International Neuropsychiatric Interview (M.I.N.I.) [44]. Individuals who meet the DSM-4 criteria for current and lifetime ADHD, as determined by the Diagnostic Interview for ADHD in adults (DIVA 2.0) [45], will be included in group (1) Individuals who meet the DSM-5 criteria for BD of any polarity, including euthymia, will be included in group (2) Group 3 consists of individuals with BPD according to the Diagnostic and Statistical Manual of Mental Disorders using the structured clinical interview for DSM-5 personality disorders (SCID-5-PD) [46], Borderline Personality Disorder part. All patient groups will receive treatment as usual, including medication, psychotherapy, and other somatic therapies, without any restrictions. The effects of therapy will be examined exploratorily in all groups and as a covariate in the main analyses. Both inpatient and outpatient treatment will be permitted as deemed necessary by the treating clinicians. Comorbid depression or anxiety disorders, as well as alcohol and cannabis use (but not meeting criteria for substance use disorder), will be allowed in all groups. Group 4 will consist of individuals who do not have any mental disorder and are not at genetic risk for ADHD, mood disorders, and psychotic disorders. Participation in other observational studies is permitted, but participation in interventional studies must be approved by the Steering Committee (SC) beforehand. The age range for participation is from 14 to 50 years of age, focusing on developmental aspects.

### Exclusion criteria

Participants will be excluded from the study if they meet any of the following criteria: pregnancy at the time of enrollment, acute suicidal thoughts at the time of enrollment, less than 6 years of regular schooling (indicative of an IQ under 75), significant neurological or mental disorders (such as schizophrenia or schizoaffective disorder, autism spectrum disorder, organic psychiatric disorder, current epilepsy, malignancies of the CNS, current or previous stroke, or early onset neurodegenerative disorder), current dependence on alcohol or illicit drugs, unwillingness to use a smartphone, and inability to read, write or provide informed consent. In addition, severe infectious disorders (such as sepsis or bacterial infection needing antibiotic treatment), severe gastrointestinal disorders, and severe vision impairment are also considered exclusion criteria. We evaluate these conditions to be severe, if they have a clinical impact on the participants’ level of activity. Furthermore, participants with a relationship to the investigator or study staff will not be eligible to participate in the study.

Written informed consent from the participants is required for inclusion. The informed consent form will be provided to all patients before the baseline assessment and in connection with the eligibility screening by the staff involved in the research project. The study complies with the principles of the revised version (last revised October 2013 by the 64th General Assembly) of the Declaration of Helsinki and the principles of the International Conference on Harmonisation - Good Clinical Practice (ICH-GCP). Participants may withdraw their consent to participate in the research at any time. This will be of no consequence. Depending on the preference expressed in the informed consent form, data recorded up to the time of withdrawal may be deleted or retained.

### Study workflow

**Fig. 2:**
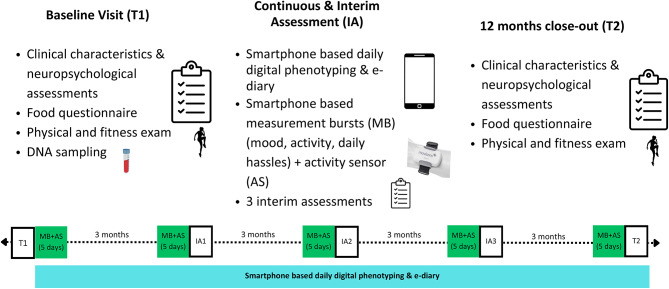
Study design: 1-year course of the study starting with the baseline visit, including clinical characteristics and neuropsychological assessments, food questionnaire, fitness tests, and blood exam for DNA extraction. This is followed by continuous and interim assessments including digital phenotyping and measurement bursts via smartphone, as well as quarterly interim assessments. The study is concluded by a close-out visit after 12 months, repeating some assessments from the baseline visit as well as the food questionnaires and fitness tests

The baseline visit (Table 2; Fig. 2) is the first step of the study. It includes validation of the participant’s already known diagnosis and a detailed assessment of BD, ADHD, and BPD symptoms using validated scales. We will also measure early traumatic life events, impulsivity, sleep, food intake, and fitness level. Additionally, we will collect a blood sample for DNA extraction and genome-wide genotyping. This will provide a comprehensive context for the high-resolution data that we will collect later.

At the baseline visit, the participants will be provided with an app that allows for e-diary assessment and digital phenotyping. This process involves collecting data from their private smartphones, assessing parameters related to communicativeness (such as phone calls and social app usage) and activity patterns (via GPS tracking). Android users will be provided with the app movisensXS (movisens GmbH, Karlsruhe, Germany), and iOS users with m-Path Sense (Katholieke Universiteit Leuven, Netherlands). During five measurement bursts distributed over the year of participation, participants will complete the e-diary on five consecutive days, starting the day after inclusion to the study (see Table 2; Fig. 2). The e-diary sends out prompts following a pseudo-random time-sampling plan at intervals of about 72 min ± 15 min, from 9 h to 21 h. Participants will receive 10 prompts per day.

The mood assessment items are a reliable tool for the ongoing measurement of temporary emotional states in research using e-diaries [47]. The conceptualization of the momentary affective state includes variation along three dimensions and participants assess their immediate emotional state by rating two opposing items on visual analogue scales for each of the following dimensions: valence (“At this moment I feel”: “content–discontent” and “unwell–well” [reverse coded]), calmness (“agitated–calm” and “relaxed–tense” [reverse coded]), and energetic arousal (“full of energy–without energy” and “tired–awake” [reverse coded]). In addition to mood, we will assess momentary self-esteem with a short form of the Rosenberg Self-Esteem Scale (4 items) [48] that was adapted and established in prior e-diary studies (for example [35]). Further items will assess impulsivity with three items validated by Halvorson et al. [49], positive and negative daily life events (two items), social interactions (two items)), and dysfunctional behavior (two items). During the five-day measurement bursts, participants will also wear wristband activity sensors (move 4, movisens GmbH, Karlsruhe, Germany) to monitor physical activity. Moreover, throughout the 365 days, the participants will complete diaries at the end of the day on mood and sleep (awake or sleepless in bed, time spent asleep during the last 24 h in 1 h segments) (adapted from ChronoRecord, which is a very well validated mood charting system [50, 51]). Once a week, the assessment of the current menstrual cycle will be included in female participants’ end-of-day diary. Each answer will be marked automatically with a time stamp by the e-diary. The prompts were translated to the individual language of the concerning site from English and are displayed for the participants as such (e.g. German for participants of the German site, Italian for the Italian site, etc.).

Additionally, the app on the participants’ smartphones will continuously collect data about their physical activity, app usage, i.e., the frequency and duration of the usage of common apps (especially social media apps), as well as incoming and outgoing calls (apps and calls only possible on Android devices). We will not assess the content of app usage and calls. The passively collected data on smartphone use enriches our e-diary data by incorporating additional context to potential digital phenotypes, e.g., with regard to communication and physical activity behavior, and will be included in our analyses to characterize temporal dynamics of affect applying the modified DynAffect model.

After each of the five measurement bursts, all questionnaires sensitive to change will be repeated via an online interim assessment (expected every 3 months until the end of the 12-month follow-up period). We will also assess the M.I.N.I. via phone to check for any clinical mood episodes. If a participant needs inpatient treatment for a depressive or manic episode, we will admit them to the according hospital for adequate care. The final, close-out visit will include the food diaries and fitness tests, as well as the scales of the interim assessment (see Table 2; Fig. 2).

Online assessments provide self-rating scales and measures of life stress (traumatic life events, daily hassles). This design allows adding environmental influences to genetics on key parameters of the modified DynAffect Model. Using such a deep phenotyping longitudinal approach in conjunction with genetic and environmental information will allow us to chart the trajectory of the disorders and correlate them to external and internal factors. Dynamic system modeling allows inferring antecedents and consequences. Such an approach combining genetics, stressor load, and longitudinal granular mobile assessment is unprecedented and will yield meaningful information on long-term courses of ADHD, BD, BPD, and their potential interplay.

**Table 2:** Schedule of visits. X, on-site; (o), on-site, only in the case of an incident, current mood episode as evidenced by M.I.N.I. affective module; I = online assessment

Measure	Baseline	3 months	6 months	9 months	Close-out
In-/exclusion crit.*	X				
Demographics	X/I				X/I
Medication*	X/I	Upon change	Upon change	Upon change	X/I
M.I.N.I.	X	Phone (affective module)	Phone (affective module)	Phone (affective module)	X
SCID-5-PD*	X				
DIVA*	X				
WURS	X/I				
ASRS	X/I	X/I	X/I	X/I	X/I
Clinical BD characteristics*	X	(o)	(o)	(o)	X
GCI-BD*	X	(o)	(o)	(o)	X
YMRS*	X	(o)	(o)	(o)	X
Altman Self Rating Mania Scale	X/I	X/I	X/I	X/I	X/I
(Alda-Scale*)	X				X
DERS	X/I				X/I
BSL-23	X/I	X/I	X/I	X/I	X/I
MEQ	X/I				
UPPS-P	X/I				X/I
PANAS	X/I	X/I	X/I	X/I	X/I
QIDS	X/I	X/I	X/I	X/I	X/I
TEMPS-A	X/I				
C-SSRS	X	(o)	(o)	(o)	X
GHQ-28	X/I	X/I	X/I	X/I	X/I
PSS	X/I	X/I	X/I	X/I	X/I
GAF*	X	(o)	(o)	(o)	X
LHC	X/I				
CTQ	X/I				
Physical exam*	X	(o)	(o)	(o)	X
Biomaterial	X	(o)	(o)	(o)	(o)
Food intake	X/I				X/I
**Digital phenotyping**	**Continuously** throughout the 12 months observational period				
**E-diary**: mood and sleep	**Daily** throughout the 12 months observational period				
**Measurement burst** (high-frequency mood dynamic)	I (5 days)	I (5 days)	I (5 days)	I (5 days)	I (5 days)

*, clinician rating/interview. Alda-Scale is only done when patients have lithium treatment > 6 months. Other interim assessments are done online. Baseline and close-out visits are done on-site. A comprehensive list of the abbreviations used is provided further down in the manuscript

### Primary experimental outcome

Our primary outcome is to test whether the dynamics of affect fluctuation as proposed by the modified DynAffect Model (homebase, affective variability, and attractor strength), differ between ADHD, BD, and BPD, and HC, using within and between measurement burst comparison.

### Secondary experimental outcomes

Owing to the rich dataset, which is generated in our project, and along the basic questions of our project, we investigate several secondary outcomes:

1. Testing the effect of stress (macro- and micro-stressors) on mood, activity, and sleep;
2. Addressing whether sleep problems precede affect fluctuations, or vice versa;
3. Testing (shared and unique) polygenic risk scores for ADHD, BD, BPD, Depression, neuroticism, and resilience for their effect on mood elation, depression, and affect fluctuation;
4. Finally, by bringing this data together, we will address the question of whether ADHD, BD, and BPD can be conceptualized to lie within a diagnostic spectrum, or whether they are overlapping but comorbid disorders.

### Sample size considerations

To enable a priori power considerations and determine a sufficient number of participants and time points for multilevel modelling, Arend and Schaefer [52] introduced a comprehensive approach using Monte Carlo simulations. Applying the proposed a priori power analyses to our planned study design, with a total of 615 possible prompts per participant over one year (level 1) in 480 individuals (level 2), and medium ICCs of our mood ratings, we estimate the power to detect small to medium effects to be greater than 0.80. These estimates are also in line with Arend and Schäfer’s rule of thumb recommendations for minimal detectable effect sizes [52].

### Statistical methods

To comprehensively analyze the three components of the modified DynAffect Model (home-base, variability, and attractor strength), we will employ established statistical approaches rooted in dynamic systems theory. This includes drawing on the original DynAffect Model [33] or the Affective Ising model proposed by Loossens et al. [39]. Specifically, to differentiate affective dynamics across the three disorders (ADHD, BD, and BPD), and between episodes (manic, depressive, remitted) within our intensive longitudinal data set, we will integrate traditional statistical metrics such as autocorrelation and mean squared successive differences. Furthermore, we will use advanced non-linear models capable of incorporating non-equidistant observations, bimodal distributions (i.e., representing different homebases), and predicting subsequent outcomes influenced by contextual factors (e.g., sleep, stress reactivity, or menstrual cycle) over time [39].

### Genetic analyses

Standard quality control procedures will be undertaken using the PLINK program. Quality control will be implemented first at the marker level, then on the individual level to maximize retention of individuals with valid data (excluding markers with less than 95% complete genotyping, invariant, or with minor allele frequency less than 1%). Hardy Weinberg Equilibrium (HWE) will be tested and controlled for. Heterozygosity and biological relatedness will be quantified using standard procedures in PLINK. Individuals will be excluded if they have genotyping completeness of less than 95%, have ambiguous genetic sex, mismatch between genotypic and phenotypic sex, or have abnormal heterozygosity on autosomes. For analyses of biological relatedness and population structure, the data will be pruned for linkage disequilibrium (LD) to remove redundant SNPs in strong LD (R^2^ > 0.6) with other SNPs and regions with known long-range LD. Biological relatedness will be assessed through identity by descent (IBD) in the LD-pruned dataset in PLINK. Genetic population structure will be mapped using an iterative principal component analysis in PLINK. External reference samples (1000 Genomes) will be used to infer the racial composition of the samples and classify subjects according to genetic heritage. Genomic information on relatedness, ethnicity, and genetic population structure will be used to control for confounding factors.

### Strategies to improve adherence to ema prompts

Improving adherence to EMA prompts in a study focused on mood variations is crucial for obtaining accurate and valuable data. We will use several strategies to enhance adherence, and by this ultimately improving the quality and reliability of data collected in the study.

1. user-friendly app: we will utilize a user-friendly EMA app that is easy to navigate and provide a seamless experience for participants; we will ensure that the interface is intuitive and visually appealing;
2. positive reinforcement: we will use a system of rewards or incentives to motivate participants, offering small incentives or personalized feedback for consistent participation, which can encourage compliance;
3. clear instructions: we will provide clear and concise instructions on how to respond to prompts and what is expected from participants, ensuring participants understand the purpose and significance of their contributions;
4. training and familiarization: we will offer comprehensive training sessions or tutorials at baseline to help participants become comfortable with the EMA app and its usage;
5. privacy and security: we will assure participants that their data are kept confidential and secure.
6. Missing data: We accounted for the completeness of smartphone data by reviewing it on a regular basis and evaluating the reason for missing data to avoid them in future prompts.

### Dissemination plans

Results of the trial will be published in peer-reviewed scientific journals, with open-access journal being prioritized. Additionally, study results will be presented at national and international scientific conferences. Results will also be disseminated to health care professionals via departmental seminars and to the general public via press and social media.

## Discussion

The present study investigates the dynamics of affect modulation and emotional dysregulation in individuals with ADHD, BPD, and BD. Emotional dysregulation describes difficulties in functional handling of changes in affect and is defined as “excessive expression and experience of emotions with rapid and poorly controlled shift in emotions and abnormal allocation of attention to emotional stimuli” [53]. Intriguingly emotional dysregulation appears transdiagnostically. In case of BPD this is even listed in the diagnostic criteria according to the DSM-V [2], in ADHD and BD this has been proved to be evident as well [53]. Here, ADHD patients showed similar levels of emotional dysregulation compared to BD patients and even higher emotional responsiveness [53].

Even though emotional dysregulation is a transdiagnostic phenomenon and affects a wealth of psychiatric patients, underlying pathomechanisms are not fully understood. From a neurobiological perspective, the brain subcortex represents the basic emotions and sends signals to various brain regions, such as the hippocampus, cingulate cortex, the insular cortex, and the amygdala [54, 55]. The forebrain structures adjust these signals. The balance between glutamate and GABA influences emotional control. The “limbic cerebellum” also participates in emotion dysregulation, and emotion dysregulation may result from congenital defects or later injuries in this area. Emotion Regulation neural circuits involve subgenual and rostral parts of the orbitofrontal, the anterior cingulate, and the dorsomedial prefrontal cortex (PFC), and regions that regulate attentional as well as executive functions, such as the dorsolateral and dorsal anterior cingulate, ventrolateral PFC [54, 56, 57].

The early onset of these disorders and their impact on daily functioning highlights the need for early identification and effective treatment. Understanding the dynamics of affect modulation in these disorders may help identify specific targets for treatment interventions. Additionally, by investigating the similarities and differences in affect regulation across these disorders, we may gain a better understanding of the underlying mechanisms and inform the development of targeted interventions.

### Study limitations

Potential limitations of the study include the small sample size and the drop-out rate due to various protocol violations. We already accounted for these issues and determined measures. To secure participants’ adherence to the study, we will contact them regularly and motivate them to stay on track with the study. The participating sites also guarantee the recruitment structure needed for such a project, as they care for a great number of patients with the investigated disorders and thereby have a great opportunity for the recruitment and supervision of such a study. Patients will benefit from participation in this study by drawing their attention to the occurrence of affective states and therefore enabling them to link their emotions to external and internal events. In the long run, patients will be empowered to detect and cope with emotional dysregulation in a more effective way leading to less symptom burden.

## Data Availability

No datasets were generated or analysed during the current study.

## Abbreviations

AA: Ambulatory Assessment
ADHD: Attention-deficit/hyperactivity disorder
Alda-Scale: Alda Scale for the Assessment of Lithium Response
ASRS: Adult ADHD Self-Report Scale
BD: Bipolar disorder
BPD: Borderline personality disorder
BSL-23: Borderline Symptom List 23
C-SSRS: Columbia-Suicide Severity Rating Scale
CTQ: Childhood Trauma Questionnaire
DERS: Difficulties in Emotion Regulation Scale
DIVA: Diagnostic Interview for ADHD in Adults
FBF: Unit of Epidemiological and Evaluation Psychiatry, Istituto di Ricovero e Cura a Carattere Scientifico (IRCCS)-St. John of God Clinical Research Centre, Brescia, Italy
GAF: Global Assessment of Functioning
GCI-BD: Global Clinical Impression for Bipolar Disorder
GHQ-28: General Health Questionnaire-28
GUF: Department for Psychiatry, Psychosomatic Medicine and Psychotherapy, University Hospital Frankfurt- Goethe University, Germany
IBD: Identity by descent
ICH-GCP: International Conference on Harmonisation - Good Clinical Practice
LD: Linkage disequilibrium
LHC: Life History Calendar
M.I.N.I.: Mini International Neuropsychiatric Interview
MEQ: Morningness-Eveningness Questionnaire
PANAS: Positive and Negative Affect Schedule
PFC: Prefrontal cortex
PRS: Polygenic risk scores
PSS: Perceived Stress Scale
QIDS: Quick Inventory of Depressive Symptomatology
SCID-5-PD: Structured clinical interview for DSM-5 personality disorders
SEM: Structure equation modelling
ST: Steering Committee
TEMPS-A: Temperament Evaluation of Memphis, Pisa, Paris, and San Diego Autoquestionnaire
UiB: University of Bergen and Division of Psychiatry, Haukeland University Hospital
UNIGE-HUG: Department of Psychiatry, Faculty of Medicine, University of Geneva, Geneva, Switzerland; TRE Unit, Division of Psychiatric Specialties, Department of Psychiatry, University Hospital of Geneva, Geneva, Switzerland
UPPS-P: Urgency-Premeditation-Perseverance-Sensation Seeking-Positive Urgency (UPPS-P) impulsive behavior scale
VHIR: Department for Psychiatry, Hospital Universitari Vall d’Hebron, Barcelona, Catalonia, Spain; Group of Psychiatry, Mental Health and Addictions, Vall d’Hebron Research Institute, Barcelona; Biomedical Network Research Centre on Mental Health, Barcelona, Spain
WURS: Wender Utah Rating Scale
YMRS: Young Mania Rating Scale
